# Stability of vitamin A at critical points in pet-feed manufacturing and during premix storage

**DOI:** 10.3389/fvets.2024.1309754

**Published:** 2024-03-04

**Authors:** Gabriela Miotto Galli, Ines Andretta, Nicolas Martinez, Bruno Wernick, Yauheni Shastak, Alvaro Gordillo, Jaqueline Gobi

**Affiliations:** ^1^Department of Animal Science, Universidade Federal do Rio Grande do Sul-UFRGS, Porto Alegre, Brazil; ^2^BASF Corp., Nutrition and Health Division, Raleigh, NJ, United States; ^3^BASF S.A., Nutrition and Health Division, São Paulo, Brazil; ^4^BASF SE, Nutrition and Health Division, Ludwigshafen am Rhein, Germany; ^5^BASF Espanola S.L., Nutrition and Health Division, Barcelona, Spain; ^6^Polinutri, Departamento Técnico, Osasco, Brazil

**Keywords:** dog, feed, processing, stability, vitamin

## Abstract

The objective of this research was to assess and quantify the potential vitamin A losses that occur during the manufacturing of pet feed and premix, as well as during their extended storage periods. This trial was conducted at a commercial feeder mill that utilized a standard commercial dog feed along with a corresponding vitamin-mineral premix. The calculated amount of vitamin A supplemented in the feed, in addition to the endogenous vitamins present in the ingredients, was adjusted to 18,000 IU/kg of feed. Five 500 g feed samples were collected at each of the predefined critical points throughout the manufacturing process (after mixing, milling, preconditioner, and extrusion/drying processes) to verify the stability of vitamin A during feed production. Additionally, various samples were collected at regular intervals of 30, 60, 90, 120, and 180 days during the storage of the premix to assess the stability of vitamin A. Vitamin A analyses in the samples were performed using high-performance liquid chromatography. The variables were assessed for normality using the Shapiro–Wilk test, followed by analysis of variance (ANOVA) and Tukey’s test to compare the differences between the manufacturing process and premix shelf life. The statistical significance was set at 95%. The vitamin losses during the pre-conditioning process were 26%, and during the extrusion-drying processes, the losses were 34% when compared to the initial analyzed value. However, no differences were observed in other processes. There were no significant differences observed in recovered vitamin levels in the premix during its shelf-life (*p* = 0.484). The study indicated that the primary vitamin A losses in pet feed manufacturing processes occur during the pre-conditioning and drying/extrusion steps. However, it is worth noting that no significant losses of vitamin A were found during the premix storage phase.

## 1 Introduction

**Figure 1:**
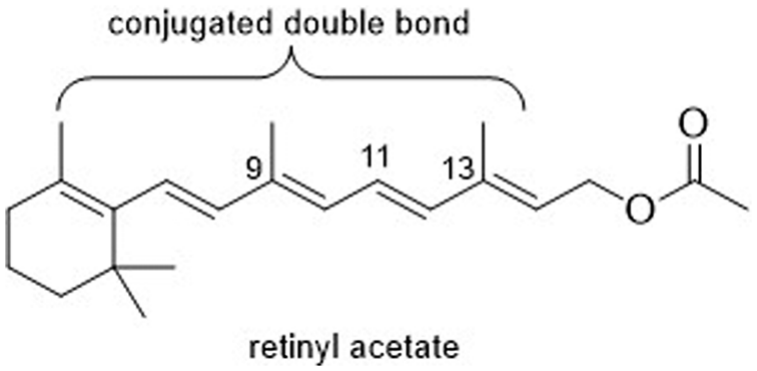
Structure of retinyl acetate.

Vitamin A is an important micronutrient required by mammals for maintaining good immune responses, epithelial integrity, body growth and development, good vision, and healthy reproductive function (1). Hypovitaminosis A leads to distortion of early offspring development, vision impairment, compromised cellular immune responses, and diminished antibody synthesis (2). This condition also induces heightened oxidative stress levels, further impacting the growth and overall well-being of animals (3). While mammals, including dogs, possess the inherent capability to convert precursor compounds, such as dietary carotenoids (e.g., β-carotene), into vitamin A, their effective capacity for such synthesis can be influenced by various factors. These factors encompass elements such as the composition of their diet, their age, and their state of health (4). Commonly, when formulating diets, the inherent levels of vitamin A and carotenoids present in feed ingredients are often overlooked due to practical considerations (2, 5). Instead, retinyl acetate is supplemented in the diet to fulfill the overall retinol requirement for animal nutrition. However, this common practice presents several challenges in the daily routine of the industry. A major concern is the potential loss of vitamin A during the manufacturing and storage processes, which can affect the final nutritional content of the feed. This issue needs to be addressed to ensure the effectiveness and feasibility of using vitamin A as a supplement in animal feed. Commercial vitamin A sources, specifically retinyl acetate (Figure 1), face challenges in terms of stability due to the presence of highly conjugated double bonds in their molecular structure. Factors such as temperature, pH, humidity, light exposure, oxygen, and other chemicals like minerals during processing and storage can adversely affect its stability (6–8).

Notably, in vitamin and mineral premixes, the high concentration of metal ions in the matrix can accelerate the degradation of vitamin A, leading to significant losses during production and storage (9). To address these issues and minimize vitamin A losses, there is a pressing need for a vitamin A feed additive that exhibits significant tolerance to these challenging conditions.

Extrusion is a vital manufacturing process that involves subjecting feed materials to elevated temperatures and pressures. This process induces physical and chemical changes in the feed ingredients, whereby the intrinsic starch undergoes gelatinization to increase digestibility and inactivates antinutritional factors (8). Furthermore, it has been observed to improve diet palatability as well as the shelf life of the final feed product (10, 11). However, extrusion has the potential to cause vitamin A degradation due to the utilization of harsh conditions and the presence of various minerals in the matrix. In light of the serious potential negative consequences arising from adverse settings, there is a lack of comprehensive research on the impact of feed manufacturing processes on micronutrient stability, particularly concerning vitamin A. Therefore, the primary objective of this study was to thoroughly investigate and measure the extent of vitamin A loss that occurs during the manufacturing of pet food and premixes. In addition, we aimed to assess the stability of vitamin A during prolonged storage.

By addressing these aspects, we aspire to gain valuable insights into the effects of the manufacturing process and storage on vitamin A content, which will contribute to a better understanding of the nutritional quality and safety of pet feeds and premixes over time. Ultimately, this research can pave the way for improved feed production practices and formulations that ensure the delivery of essential nutrients to pets, thereby promoting their overall health and well-being.

## 2 Materials and methods

### 2.1 Diet formulation, extrusion processing, and sample collection

A commercial feed was formulated to meet the nutritional maintenance requirements for adult dogs (12) for use in this trial (Table 1). The vitamin-mineral premix for dogs was also formulated considering the requirements of adult dogs (Table 2) and added to the feed at the inclusion rate suggested by the manufacturer. The vitamin A content in the feed was determined to be 18,000 IU/kg. The study trial was conducted in a commercial feed mill with a daily production capacity of approximately 130 mt feed. All the samples were collected from a single production batch. The vitamin A feed additive used in the trial was a beadle product manufactured by BASF, which utilized microencapsulation technology (BASF SE, Ludwigshafen am Rhein, Germany).

**Table 1:** Nutritional composition of the feed.

Ingredients	Inclusion, %
Rice grits	42.27
Meat meal	20.78
Poultry fat	9.44
Swine protein	9.20
Wheat gluten feed	2.00
Dehydrated hydrolyzed yeast	4.00
Dicalcium phosphate	1.00
Limestone	1.50
Fish oil	1.00
Beet pulp	4.00
Liver palatabilizer	2.50
Premix	2.00
Salt	0.31

	Calculated values
Crude protein (g/kg)	240
Crude fat (g/kg)	120
Crude fiber (g/kg)	25
Total calcium (g/kg)	12
Total phosphorus (g/kg)	7
Sodium (g/kg)	2
Vitamin A (UI/kg)	18,000

**Table 2:** Nutritional composition^1^ of vitamin-mineral premix.

	Calculated values
Iron (mg)	3,600
Copper (mg)	1,000
Manganese (mg)	7,000
Zinc (g)	12
Iodine (mg)	200
Selenium (mg)	40
Chromium (mg)	18
Vitamin A (UI)	1,800,000
Vitamin D3 (UI)	80,000
Vitamin E (UI)	50
Vitamin K3 (UI)	400
Vitamin B1 (UI)	500
Vitamin B2 (UI)	1.600
Vitamin B6 (UI)	500
Vitamin B12 (UI)	15,000
Vitamin C (g)	20
Niacine	10
Calcium pantothenate (mg)	2,000
Folic acid (mg)	150
Biotine (mg)	40

^1^Provided per kg.

In the production of vitamin-mineral premix, the respective ingredients were manually weighed and charged into a double-helix horizontal ribbon mixer with 900 kg capacity (Máquinas Ferraz, Ribeirão Preto, Brazil). The ingredients were mixed thoroughly for 4 min. The premix was then bagged (closed paper bags) and stored in a controlled temperature room (at 5°C) until further use or sampling.

The feed ingredients were ground in a hammer mill (Máquinas Ferraz, M1000 and M700 models, Ribeirão Preto, Brazil) fitted with a 0.8 mm size screen sieve operating at a throughput of 6 tons/h. After the particles were reduced to the desired size, samples were collected from five specific points (experimental units) in the mixture. These samples were used to determine the concentration of the vitamin A present. The feed production process involved mixing the ground raw materials in a double-helix horizontal mixer for a duration of 5 min. The mixer utilized was a Máquinas Ferraz model from Ribeirão Preto, Brazil, with a capacity of 1.6 mt and equipped with a 50 HP power motor. Additionally, a mixer was set up for automatic injection of liquid soybean oil, lysine, and methionine into the mixture. This streamlined approach allowed for efficient and precise feed production.

The feed was first treated in a non-pressurized preconditioner (Máquinas Ferraz, Ribeirão Preto, Brazil) with steam and water injection. This process lasted for 90 s and operated at a temperature of 90°C with a humidity level of approximately 22%. Next, the conditioned feed was transferred to a single-thread extruder (Máquinas Ferraz, Model E240, Ribeirão Preto, Brazil) designed to handle 10 mt of feed per hour. The extruder’s final processing temperature ranged from 90°C to 100°C (before the cooling process). The humidity level was reduced to about 6.5%, and the pressure during extrusion was maintained at 250 amperes. The extruder’s cutting knife speed at the end of the process was set at approximately 42 rpm. Following extrusion, the feed underwent a double-pass horizontal dryer for further processing. From the dryer, the feed was directed to a pneumatic exhaust with a rotary valve to be spread evenly on a conveyor. This formed a 12 cm high layer in which the feed remained for a total of 18 min: 8 min on the upper conveyor, where the first contact with heat occurred, and then 10 min on the lower conveyor, where moisture was removed. The feed was initially directed to a conveyor, leading to a vibrating sieve, which had two separate sections with sieves of sizes 4 mm and 14 mm. After passing through this sieve system, the feed’s mold diameter was reduced to 8.1 mm. Consequently, the final dimensions of the processed feed were approximately 12–14 mm in diameter and 5–7 mm in thickness.

### 2.2 Sampling during feed processing and premix storage

To verify the stability of vitamin A during the feed production process, five 500 g feed samples were collected at specific critical points: after mixing, milling, preconditioner, and extrusion/drying processes. Additionally, samples of premix were gathered at regular intervals of 30, 60, 90, 120, and 180 days to assess vitamin A stability during the premix storage period used for the pet feed. After collection, all samples were carefully bagged, properly labeled, and stored at a controlled temperature of 5°C. This storage condition ensured the preservation of the samples for accurate analysis and evaluation.

### 2.3 Laboratory analysis

The analysis of vitamin A in various samples utilized high-performance liquid chromatography (HPLC). To achieve uniformity, the samples were first ground to a 1 mm consistency. Subsequently, hydrolysis was conducted using an ethanolic potassium hydroxide solution to effectively break down the compounds. Finally, extraction into light petroleum (BSN) was performed to separate the all-trans-vitamin A alcohol from its cis isomers.

### 2.4 Statistical analysis and interpretation

Data was collected in a completely randomized design with five replications for each processing step under study. Grubbs test was used to detect eventual outliers in each sampling point, but no atypical values were identified. The data were then subjected to variance analysis using the General Linear Model procedure (the fixed effect of the points in pet feed manufacturing was considered for feed, while the storage time was considered for premix), followed by Tukey’s test to detect eventual differences among treatments at a 95% confidence level. Regression fitting (linear and quadratic) was also tested to assess the vitamin recovery in the premix over the storage time. All analyses were performed using the Minitab 20 software (Minitab Inc., State College, PA, United States).

Data interpretation involved comparing the vitamin A activity observed at each critical point to the desired levels (targeting 18,000 IU in the feed and 1,800,000 IU in the premix). Furthermore, the observed (analyzed) vitamin A levels at the beginning of each test (for both the feed mixture and newly produced premix) were taken into account. Additionally, vitamin A activity was compared at various critical points during the feed milling process and premix shelf-life assessment.

## 3 Results

**Figure 2:**
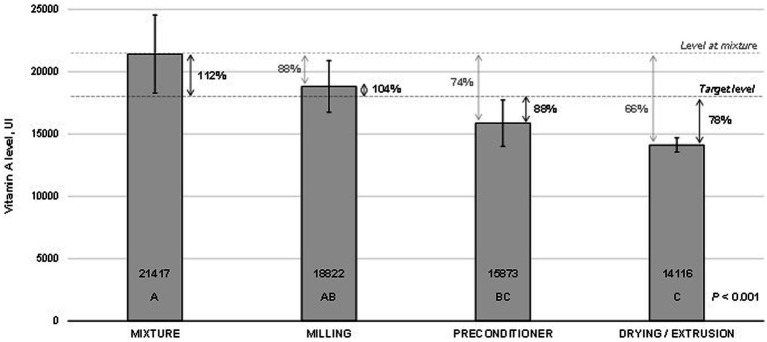
Stability^1^ of vitamin A across critical points in pet feed manufacturing. ^1^Bars indicate the means and standard deviation obtained from five samples collected at each data point. The means were compared using variance analysis (*p* < 0.001). Grouping information is represented by letters, and these groups were established using the Turkey method with a 95% confidence level. Means that do not share a letter are considered significantly different (*p* < 0.05).

**Figure 3:**
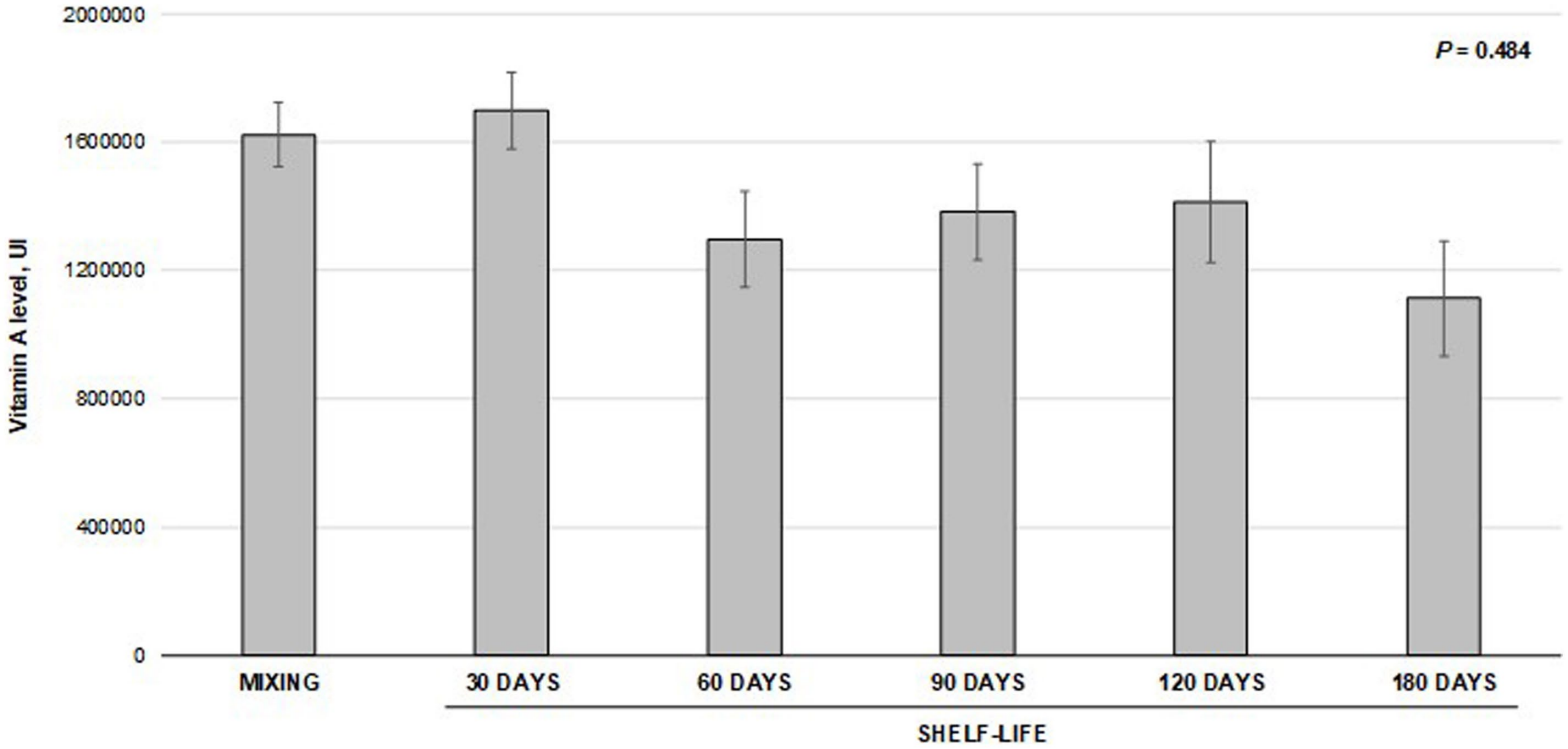
Stability of vitamin A in premix during shelf-life storage^1^. ^1^Bars indicate the means and standard deviation obtained from five samples collected at each data point. The means were compared using variance analysis (*p* = 0.484).

The vitamin A levels measured at critical points through the pet feed manufacturing are presented in Figure 2. The results did not differ between mixing and milling processes. However, vitamin A levels measured at the preconditioner and extrusion-drying stages were lower (*p* < 0.001) than those obtained in the mixture. The levels observed at the preconditioner accounted for 74% of those achieved at the initial evaluation point (mixture) and 88% of the target level (18,000 IU/kg). Similarly, the levels observed during extrusion drying accounted for 66% of those obtained at the mixture stage or 78% of the target level. Moreover, the values obtained with the preconditioner were statistically comparable to those observed during extrusion-drying. The coefficient of variation among the samples consistently decreased throughout the manufacturing process. It began at 13% during the mixture stage and gradually decreased to approximately 10% during milling and preconditioning. Ultimately, it reached an impressive 3.6% at the extrusion-drying stage. Despite the observed reduction in vitamin A recovery during feed production, there were no significant differences in the measured vitamin levels throughout the shelf-life of the premix (*p* = 0.484; Figure 3). Regression fitting (linear and quadratic) was also not significant (*p* > 0.05) for the vitamin recovery during premix storage (vitamin levels vs. storage time). The assessments were conducted for a period of up to 180 days, which is a standard shelf-life duration for this type of product.

## 4 Discussion

Environmental factors, such as temperature, oxidation, abrasion, and humidity can significantly affect the stability of vitamins (8). Among these factors, vitamin A loss is mainly influenced by oxidation, light exposure, humidity, temperature, minerals (copper, zinc, and iron), and lipid peroxidation (13). Thus, it is crucial to understand the retention rates of vitamins throughout the entire feed manufacturing process until the final consumption stage. This information is of utmost importance to the industry, as it enables them to compensate for any potential losses during processing and storage, thereby preventing both under- and oversupply of vitamins.

The molecule of vitamin A contains both free hydrogen groups and five double bonds. When retinol undergoes esterification with acetic acid during the production of commercial vitamin A sources, it forms a retinol ester that is less susceptible to oxidation. However, it is not sufficiently stable without a specific formulation (8). Therefore, to enhance its stability encapsulation is one of the alternatives, it’s emulsifying retinol with gelatin and sugars and including an antioxidant in the formulation.

Baller (14) described a 36% loss of vitamin A during the milling process. The milling losses could be attributed to the increased temperature and continuous flow of oxygen during this process (15). In the present study, significant losses were observed during pre-conditioning, amounting to 26% compared to the initial evaluation point. Additionally, an extra 8% loss occurred after the drying/extrusion stage. These losses are likely attributed to oxidative effects caused by the high temperatures employed during the drying process (90°C–100°C). Furthermore, several other factors in this process, such as moisture, friction, and pressure, may pose challenges for vitamins, such as A, C, D, and E vitamins.

In contrast to the results reported by Baller (14), our study found no significant differences in vitamin A activity during the pre-conditioning and extrusion steps. This discrepancy could be attributed to the variation in the vitamin A sources evaluated in both studies. Baller (14) highlighted that the drying process was the primary factor responsible for vitamin A loss, accounting for 42% of the total loss. They further concluded that the final retention of vitamin A was approximately 38.5% lower than that obtained before grinding the ingredients. In contrast, Yang et al. (8) discovered that the main contributors to vitamin A losses during the extrusion process are the pressure exerted on the die and high temperature. These factors led to losses exceeding 50%. The authors suggested that lowering the temperature and pressure of the extruder could help mitigate vitamin degradation. However, the specific optimal values for the pressure and temperature to prevent these losses remain unknown. Applying a fat coating containing fat-soluble vitamins to extruded feed is a practical option to avoid this damage, however, it is not frequently used in feeding mills. Loss of vitamin activity in both the premix and feed during storage can lead to reduced growth and nutritional efficiency in animals, increasing the risk of vitamin deficiency-related diseases in various species (16).

Several factors can impact the stability of vitamins in premixes and feeds during pelleting and storage, such as temperature, humidity, conditioning time, oxidation-reduction (redox) reactions, and exposure to light. The different methods used for feed processing can cause significant variations in heat, pressure, humidity, friction, and redox reactions (17). Furthermore, the presence of choline chloride and high concentrations of copper and zinc are known to influence the stability of vitamins in premixes (18). Choline chloride increases the humidity of the premix because of its hygroscopic properties, and copper and zinc can catalyze the generation of free radicals, thereby oxidizing antioxidants during storage (18). Hence, it is also crucial to assess and quantify vitamin A losses in the premix during storage.

Despite identifying a numerical change of 30% in the stability of vitamin A in the premix over various storage periods, we did not observe any statistical difference. Yang et al. (8) discovered that storing vitamin premixes for 12 months resulted in a mere 4% loss of vitamin A concentration. The authors proposed that this minimal loss could be attributed to the hydroxy group of vitamin A, which is protected by forming an ester, reducing its vulnerability to oxidation. Furthermore, Yang et al. (19) also linked the limited impact of storage time on vitamin stability to microencapsulation treatment, which likely shields the vitamins from chemical reactions. The disparity between our findings and those of Yang et al. (19) regarding vitamin A degradation could potentially be elucidated by considering variations in the type of premix used (dog versus piglet), divergent preparation, and storage conditions. It is important to acknowledge the potential influence of disparities in analytical methodologies that should not be overlooked. Moreover, the permissible range for retinol analysis, as outlined in the VDLUFA (20) guidelines, allows for a variability of around 20%–30%. Ultimately, the numerical decline observed in our experimental trial did not exhibit statistically significant differences when compared with the initial baseline measurement.

The number of research groups that specialize in feed mill processing is relatively small, even though this industry is dynamic and involves numerous interacting factors. The current research evaluated the issue of vitamin A loss in a typical industrial environment. However, additional research, coupled with a greater volume of data, is crucial for gaining a more profound comprehension of the nutrient loss patterns. Data-driven decisions are the most effective way to reduce vitamin A loss and also to enhance other aspects of feed manufacturing.

## 5 Conclusion

The most significant vitamin A losses in pet feed manufacturing processes occur during the pre-conditioning stage and the drying/extrusion stage. However, it is worth noting that there were no significant losses of vitamin A during the premix storage.

## Data availability statement

The raw data supporting the conclusions of this article will be made available by the authors, without undue reservation.

## Author contributions

GG: Data curation, Formal analysis, Methodology, Project administration, Writing – original draft, Writing – review & editing. IA: Data curation, Formal analysis, Funding acquisition, Methodology, Project administration, Supervision, Validation, Visualization, Writing – original draft, Writing – review & editing. NM: Conceptualization, Data curation, Formal analysis, Funding acquisition, Investigation, Methodology, Project administration, Supervision, Validation, Writing – original draft, Writing – review & editing. BW: Conceptualization, Data curation, Formal analysis, Funding acquisition, Investigation, Methodology, Project administration, Supervision, Validation, Writing – original draft, Writing – review & editing. YS: Data curation, Formal analysis, Funding acquisition, Methodology, Project administration, Supervision, Validation, Visualization, Writing – original draft, Writing – review & editing. AG: Data curation, Formal analysis, Funding acquisition, Methodology, Project administration, Supervision, Validation, Visualization, Writing – original draft, Writing – review & editing. JG: Conceptualization, Formal analysis, Investigation, Methodology, Validation, Writing – review & editing.

## References

[1] Soares MM Silva MA Garcia PPC Da Silva LS Da Costa GD Araújo RMA. Effect of vitamin A supplementation: a systematic review. Ciênc Saúde Colet. (2019) 24:827–38. doi: 10.1590/1413-81232018243.07112017, PMID: 30892504

[2] Shastak Y Pelletier W. The role of vitamin A in non-ruminant immunology. Front Anim Sci. (2023) 4:1197802. doi: 10.3389/fanim.2023.1197802

[3] Shastak Y Gordillo A Pelletier W. The relationship between vitamin A status and oxidative stress in animal production. J Appl Anim Res. (2023) 51:546–53. doi: 10.1080/09712119.2023.2239319

[4] Green AS Fascetti AJ. Meeting the vitamin A requirement: the efficacy and importance of β-carotene in animal species. Sci World J. (2016) 2016:7393620. doi: 10.1155/2016/7393620, PMID: 27833936 PMC5090096

[5] Darroch CS. Vitamin A In: Lewis AJ Southern LL, editors. Swine nutrition. New York: CRC Press (2000). 263–80.

[6] Loewen A Chan B Li-Chan ECY. Optimization of vitamins A and D3 loading in re-assembled casein micelles and effect of loading on stability of vitamin D3 during storage. Food Chem. (2018) 240:472–81. doi: 10.1016/j.foodchem.2017.07.126, PMID: 28946300

[7] Riaz MN Asif M Ali R. Stability of vitamins during extrusion. Crit Rev Food Sci Nutr. (2009) 49:361–8. doi: 10.1080/10408390802067290 19234945

[8] Yang P Wang H Zhu M Ma Y. Evaluation of extrusion temperatures, pelleting parameters, and vitamin forms on vitamin stability in feed. Animals. (2020) 10:894. doi: 10.3390/ani10050894, PMID: 32443930 PMC7278472

[9] Athar N Hardacre A Taylor G Clark S Harding R Mclaughlin J. Vitamin retention in extruded food products. J Food Compos Anal. (2006) 19:379–83. doi: 10.1016/j.jfca.2005.03.004

[10] Moscicki L. (2010) Extrusion-cooking techniques: applications, theory and sustainability. Nova Jersey, EUA: Wiley.

[11] Gibson M Alavi S. Pet food processing—understanding 1048 transformations in starch during extrusion and baking. Cereal Foods World. (2013) 58:232–6. doi: 10.1094/CFW-58-5-0232

[12] FEDIAF. (2019). Nutricional guidelines. Available at: https://oehtv.at/fileadmin/pdf-Dateien/2019_FEDIAF_Nutritional_Guidelines.pdf. (Accessed July 20, 2023).

[13] Charlton SJ Ewing WN. The vitamin directory. England: Context Products Ltd. (2007).

[14] Baller MA. Particle reduction, mechanical energy transference and drying on vitamins a, B2 and B6 retention in extruded dog foods In: Doctoral thesis. Jaboticabal: UNESP (2020). 93–112.

[15] Camire ME. (2001). Extrusion and nutritional quality. Extrusion cooking, technology and application, Edited by: Guy R. 1st edition, Boca Raton, Florida: CRC Press. 116–118.

[16] Shurson GC Salzer TM Koehler DD Whitney MH. Effect of metal specific amino acid complexes and inorganic trace minerals on vitamin stability in premixes. Anim Feed Sci Technol. (2011) 163:200–6. doi: 10.1016/j.anifeedsci.2010.11.001

[17] Coelho M. Vitamins and carotenoids in pet care In: Kvamme JL Phillips TD, editors. Petfood technology. Morris, IL: Watt Publishing Company (2003)

[18] Yang P Wang HK Zhu M Li LX Ma YX. Degradation kinetics of vitamins in premixes for pig: effects of choline, high concentrations of copper and zinc, and storage time. Anim Biosci. (2021) 34:701–13. doi: 10.5713/ajas.20.0026, PMID: 32810935 PMC7961278

[19] Yang P Wang H Zhu M Ma Y. Effects of choline chloride, copper sulfate and zinc oxide on long-term stabilization of microencapsulated vitamins in premixes for weanling piglets. Animals. (2019) 9:1154. doi: 10.3390/ani9121154, PMID: 31888146 PMC6941071

[20] VDLUFA (Verband Deutscher Landwirtschaftlicher Untersuchungs- und Forschungsanstalten). (2018). Analysenspielräume für Futtermitteluntersuchungen. https://www.vdlufa.org/Dokumente/Fachgruppen/FG6/ASR_Version_11_2018.pdf. (Accessed August 22, 2023).

